# COVID-19 impact on HIV PrEP uptake and retention at selected health facilities in Eswatini

**DOI:** 10.4102/phcfm.v16i1.4685

**Published:** 2024-12-19

**Authors:** Musa B. Ginindza, Nondumiso Ncube, Renier Coetzee

**Affiliations:** 1 School of Public Health, Faculty of Community and Health Sciences, University of the Western Cape, Cape Town, South Africa

**Keywords:** primary care, HIV, pre-exposure prophylaxis, COVID-19, Eswatini, retention, pandemic, public health, prevention

## Abstract

**Background:**

Oral pre-exposure prophylaxis (PrEP) uses antiretroviral medication to reduce HIV risk in HIV-negative individuals. Despite its effectiveness, global uptake faces policy and accessibility challenges. In Eswatini, PrEP introduction in 2017 showed promise despite stigma and COVID-19 disruptions.

**Aim:**

This study compared PrEP uptake and retention during and after COVID-19.

**Setting and Methods:**

An analytical cross-sectional study was conducted among clients accessing HIV testing services in selected Eswatini facilities. Data from the HIV testing register, PrEP register, and Client Management Information System (CMIS) were analysed. Uptake, retention, and client outcomes were measured during COVID-19 (March 2020–March 2021) and post-COVID-19 (April 2021–April 2022).

**Results:**

Of 5286 clients, 45% (*n* = 2380) initiated PrEP during COVID-19, while 55% (*n* = 2906) initiated post-pandemic. Facility 3 had the highest initiations during COVID-19 (844), while Facility 5 had the lowest (7). Retention was lower among clients aged 15–29 years. Females initially showed higher retention odds (odds ratio [OR]: 1.50), but this was insignificant after adjusting for confounders. Clients initiated post-COVID-19 had higher retention odds (OR: 2.96).

**Conclusion:**

COVID-19 impacted PrEP uptake in Eswatini, emphasising the need for flexible healthcare delivery. Targeted campaigns and tailored interventions are crucial for sustaining HIV prevention efforts and addressing demographic shifts.

**Contribution:**

This study highlights the importance of responsive healthcare systems and tailored approaches to maintaining HIV prevention during public health crises.

## Introduction

Oral pre-exposure prophylaxis (PrEP) is the use of antiretroviral medication by individuals who are human immunodeficiency virus (HIV)-negative to reduce the risk of acquiring HIV.^1,2^ The World Health Organization (WHO)^3^ recommends that people at high risk of contracting HIV be offered antiretroviral medication, as an additional option, as part of comprehensive prevention.^2^ The 2019 UNAIDS Global AIDS Update reported that, globally, there were an estimated 37.9 million (32.7–44.0 million) people living with HIV, with 1.7 million (1.4–2.3 million) new infections and 770 000 (570 000 to 1.1 million) acquired immunodeficiency syndrome (AIDS)-related deaths in 2018.^4^ Compared to 2010, HIV incidence at the global level has declined by 16%, whereas estimates of AIDS-related deaths have dropped by 33%; the latter is largely attributable to treatment scale-up.^5^ Despite a decrease in HIV incidence, the global HIV pandemic continues to burden the world with new infections exceeding the projected 500 000 per annum.^5^ The 2019 UNAIDS report showed that gains in reducing HIV deaths and curtailing new infections in Eastern and Southern Africa were driving global progress around 2018.^5^

To achieve HIV epidemic control, comprehensive HIV prevention efforts are needed. Globally, various prevention interventions to reduce and stop the spread of HIV have been implemented, with oral PrEP being one of the interventions.^1^ Pre-exposure prophylaxis, defined as the use of antiretroviral medication to prevent HIV acquisition among at-risk persons, is an effective HIV prevention method.^6^ Pre-exposure prophylaxis programmes have slowly scaled up in some countries because of policy and accessibility barriers. In 2016, PrEP Watch reported that only nine countries had initiated approximately 100 000 persons on PrEP; four were in Africa: Ethiopia, Senegal, South Africa, and Zimbabwe.^6^ Thus, the total number of people who have been enrolled on PrEP has fallen short of the UNAIDS goal of three million persons on PrEP by 2020.^6^

In Eswatini, oral PrEP was introduced in 2017, mainly targeted at populations at a higher risk of HIV acquisition.^7^ Pre-exposure prophylaxis was introduced as part of the healthcare services provided for family planning, antenatal care, outpatients, and HIV testing sites.^8^ In Eswatini, 28 575 people were enrolled in PrEP in 2021.^7^ In 2017, there were 415 new users. This number increased to 1278 new users in 2018, and 7809 new users in 2021.^7^ Since the programme’s inception, uptake of oral PrEP has been substantial despite the associated stigma, such as concerns that others might assume users are HIV-positive and undergoing antiretroviral treatment.^9^

Oral PrEP is highly effective in preventing HIV infection if used as directed. The roll-out of PrEP is expanding worldwide, including across sub-Saharan Africa.^10^ In generalised epidemic settings, strategies are needed to identify and engage individuals who might benefit from HIV prevention services, including PrEP.^11^ Pre-exposure-prophylaxis has been heralded for its potential to put people at risk of contracting HIV in control of preventing HIV infection. Significant strides made in HIV epidemic control, such as the scale-up of antiretroviral therapy, were threatened by the coronavirus disease 2019 (COVID-19), caused by severe acute respiratory syndrome coronavirus 2 (SARS CoV-2).^12^

Numerous studies have documented significant changes in the usage of healthcare services during the COVID-19 pandemic because of measures such as lockdowns and stay-at-home orders implemented to curb the spread of the virus.^13^ These COVID-19 restrictions negatively impacted healthcare service provision, particularly in areas severely affected by the pandemic.

A WHO survey reported that disruption to healthcare services was greatest among lower-income countries.^14^ Many people could not access treatment and vaccinations that they desperately needed, such as cancer treatments that extended their lives.^15^ Although there were disruptions in healthcare service provision, COVID-19 restrictions positively introduced and strengthened other healthcare services, such as telemedicine.^12^ Concurrently, the pandemic may also have resulted in some people being spared unnecessary or inappropriate care, which has the potential to cause harm.^14^

Eswatini has one of the highest HIV prevalence rates globally; 27% of adults are HIV-positive. The HIV and/or AIDS pandemic remains the country’s most significant public health threat. To combat this, Eswatini has implemented various strategies, including antiretroviral therapy (ART) for HIV-positive individuals and PrEP for HIV-negative individuals.^16,17^ This study aimed to describe the effects of COVID-19 on PrEP uptake and retention at selected health facilities in Eswatini.

## Methods

This study was conducted in five selected public health facilities in Eswatini. Eswatini, a lower middle-income country in Southern Africa with a population of about 1.1 million, is landlocked by South Africa and Mozambique.^2^ The country is divided into four regions: Hhohho, Manzini, Shiselweni, and Lubombo. Manzini, the industrial hub, is the most populous region with approximately 355 945 residents, and this region attracts youth from the other regions for employment and education. The selected facilities are in the Manzini region.^18^ The facilities were selected through a non-probability sampling method in which a regional referral facility (facility 5) was chosen together with its baby clinics where PrEP was piloted and scaled up. Table 1 describes the characteristics of the selected facilities.

**TABLE 1:** Facility characteristics.

Name of facility	Characteristics of facility	Region
Facility 1	A public health facility located in a densely populated township next to a regional referral hospital	Manzini
Facility 2	An ART facility in the Manzini town city centre	Manzini
Facility 3	A clinic located in a highly populated area next to a university	Manzini
Facility 4	A public health facility located in the industrial hub of the country. This facility has flexi-hours and is male-friendly	Manzini
Facility 5	A referral hospital located at the centre of the region	Manzini

ART, antiretroviral therapy.

An analytical cross-sectional study design with a quantitative approach was utilised to ascertain PrEP uptake and retention during (March 2020–March 2021) and after (April 2021–April 2022) COVID-19. Pre-exposure prophylaxis uptake and retention were reviewed during these two time points. The dependent variable was PrEP uptake and retention rate, and COVID-19 was the exposure or independent variable. Routinely collected programme data on PrEP uptake and retention in care were extracted from the electronic medical records (the client management information system [CMIS]) and registers in the selected facilities. The five facilities were selected for the study because they have high patient volumes.

The study population included clients who accessed HIV testing services at these selected facilities. Participants who tested negative for HIV and started PrEP between March 2020 and March 2021 during COVID-19 and April 2021 and April 2022 after COVID-19 were included in the study. Eligible clients included individuals aged 16 years and older, as well as sexually active mature minors under 16, prescribed PrEP in accordance with the Eswatini Integrated HIV Management Guidelines.^7^ Clients eligible for PrEP in Eswatini must have no contraindications to tenofovir disoproxil fumarate (TDF) plus lamivudine (3TC), no signs of acute HIV infection (AHI), be willing to adhere to PrEP visits, have a body weight over 30 kg, and a creatinine clearance above 60 mL/min.^7^ In addition, clients registered as those belonging to the selected facilities, that is not visiting the facilities to refill PrEP are also eligible. However, clients excluded were those who were started on PrEP in other facilities and transferring in from other facilities.

### Data analysis

Data were analysed using STATA version 18. Socio-demographic characteristics of PrEP clients were summarised using descriptive statistics, with means and standard deviations reported for normally distributed numerical variables such as age. Multinomial logistic regression was employed to determine factors associated with PrEP initiation during and after COVID-19 and also to determine factors associated with PrEP retention, which was the main objective of the study. Assumptions for multinomial logistic regression were thoroughly checked, including linearity of logits, independence of irrelevant alternatives using the Hausman-McFadden test, and multicollinearity using the variance inflation factor (VIF). Univariable analysis was initially conducted, including all variables with a *p*-value below 0.25, followed by a manual backward elimination stepwise procedure to exclude variables with *p*-values exceeding 0.05. Crude and adjusted odds ratios, 95% confidence intervals (CI), and *p*-values were calculated for each independent variable.

### Ethical considerations

Ethical clearance to conduct this study was obtained from the University of the Western Cape Faculty of Community and Health Science Biomedical Ethics Research Committee (No. BM22/10/2).

## Results

### Socio-demographic factors of pre-exposure prophylaxis clients in Eswatini

A total of 5286 client records were analysed. Out of the 5286 individuals initiating PrEP, 45% (*n* = 2380) of clients were during COVID-19, while 55% (*n* = 2906) were after COVID-19. Facility 3 had the highest number of initiations (844), while Facility 5 had the least PrEP initiations^6^ during the COVID-19 pandemic. However, post-COVID-19, Facility 1 had the highest PrEP initiations (1634), while Facility 5 had the least PrEP initiations (30).

Most of the clients who initiated PrEP during COVID-19 were aged between 30 and 39 years (59.0%; *n* = 850), while those aged between 15 and 19 years were the least (25.5%; *n* = 152). Among the clients who initiated PrEP after COVID-19, most were aged 20–24 years (71.5%, *n* = 959), while those aged 40 years and above were the least (26.3%; *n* = 191). More female (61.6%, *n* = 1634) than male clients were initiated on PrEP during COVID-19. After COVID-19, the proportion of females initiated on PrEP increased from 40.1% (*n* = 1634) to 59.9% (*n* = 2441). For males, 61.6% (*n* = 746) were initiated during COVID-19, with a decrease to 38.4% (*n* = 465) after COVID-19. More than 70.7% (*n* = 198) of high-risk males (30–34 years) were initiated during COVID-19, but this proportion reduced to 29.3% (*n* = 82) after COVID-19. First initiations were the most common, representing 1918 (88.2%) of initiations during COVID-19 and 2361 (95.6%) after COVID-19 (see Table 2).

**TABLE 2:** Demographic characteristics of the clients (*N* = 5286).

Variables	PrEP initiation period				*N*	%
	During COVID-19†		After COVID-19‡			
	*n*	%	*n*	%		
**Facility name**						
Facility 1	767	31.9	1634	68.1	2401	100.0
Facility 2	511	63.6	292	36.4	803	100.0
Facility 3	844	48.4	901	51.6	1745	100.0
Facility 4	251	86.7	49	16.3	300	100.0
Facility 5	7	18.9	30	81.1	37	100.0
Total	2380	45.0	2906	55.0	5286	100.0
**Age group (years)**						
15–19	152	25.5	445	75.5	597	100.0
20–24	383	28.5	959	71.5	1342	100.0
25–29	460	39.0	720	61.0	1180	100.0
30–39	850	59.9	591	41.0	1441	100.0
40 and above	535	73.7	191	26.3	726	100.0
Total	2380	45.0	2906	55.0	5286	100.0
**Gender**						
Female	1634	40.1	2441	59.9	4075	100.0
Male	746	61.6	465	38.4	1211	100.0
Total	2380	45.0	2906	55.0	5286	100.0
**Population type**						
High-risk males (30–34 years)	198	70.7	82	29.3	280	100.0
Adolescent girls and young women (16–24 years)	435	26.0	1237	74.0	1672	100.0
Total	633	32.4	1319	67.6	1952	100.0
**Initiation type**						
First initiation	1918	44.8	2361	55.2	4279	100.0
Restart less than 12 months	299	79.3	78	20.7	377	100.0
Restart after 12 months	38	79.2	10	20.8	48	100.0
Transfer In	125	85.0	22	15.0	147	100.0
Total	2380	49.1	2471	50.9	4851	100.0

PrEP, pre-exposure prophylaxis; COVID-19, coronavirus disease 2019.

† , (*n* = 2380: 45%);

‡ , (*n* = 2906: 55%).

### Factors associated with pre-exposure prophylaxis initiation during the COVID-19 period as compared to after COVID-19

Out of the 5286 clients included in the study, 45% (*n* = 2380) were initiated on PrEP during the COVID-19 period, while 55% (*n* = 2906) were initiated after COVID-19. Compared to Facility 1, Facility 2 (adjusted odds ratio [aOR]: 2.58, 95% confidence interval [CI]: 2.09–3.18, *p* < 0.001) and Facility 3 (aOR: 1.95, 95% CI: 1.70–2.25, *p* < 0.001) had higher odds of initiating clients on PrEP during COVID-19. Individuals aged 25–29 (aOR: 1.55, 95% CI: 1.23–1.96, *p* < 0.001), 30–39 (aOR: 3.29, 95% CI: 2.61–4.13, *p* < 0.001), and 40 years and above (aOR: 6.36, 95% CI: 4.80–8.44, *p* < 0.001) had significantly higher odds of starting PrEP during COVID-19. Males had 2.40 times higher odds of initiating PrEP during COVID-19 (odds ratio [OR]: 2.40, 95% CI: 2.10–2.73, *p* < 0.001) compared to the females; however, the association was not significant after adjusting for potential confounders (Table 3).

**TABLE 3:** Factors associated with the pre-exposure prophylaxis initiation period among the clients.

Variables	PrEP initiation period†		Bivariate analysis			Multiple logistic		
	*n*	%	OR	95%CI	*p*	aOR	95%CI	*p*
**Facility name**								
Facility 1	767	32.0	Ref	Ref	-	-	-	-
Facility 2	511	63.4	3.73	3.15–4.41	< 0.001	2.58	2.09–3.18	< 0.001
Facility 3	844	48.4	2.00	1.76–2.27	< 0.001	1.95	1.70–2.25	< 0.001
Facility 4	251	83.7	10.91	7.94–15.00	< 0.001	-	-	-
Facility 5	7	18.9	0.50	0.22–1.14	0.098	-	-	-
**Age category (years)**								
15–19	152	25.5	Ref	Ref	-	-	-	-
20–24	383	28.5	1.17	0.94–1.46	0.162	-	-	-
25–29	460	39.0	1.87	1.50–2.33	< 0.001	1.55	1.23–1.96	< 0.001
30–39	850	59.0	4.21	3.41–5.20	< 0.001	3.29	2.61–4.13	< 0.001
40 and above	535	73.7	8.20	6.40–10.50	< 0.001	6.36	4.80–8.44	< 0.001
**Gender**								
Female	1634	40.1	Ref	Ref	-	-	-	-
Male	746	61.6	2.40	2.10–2.73	< 0.001	-	-	-
**Population type**								
Adolescent girls and young women (16–24 years)	435	26.0	Ref	Ref	-	-	-	-
High-risk males (30–34 years)	198	70.7	6.87	5.19–9.08	< 0.001	-	-	-
**Initiation type**								
First initiation	1918	44.8	Ref	Ref	-	-	-	-
Restart less than 12 months	299	79.3	4.72	3.65–6.10	< 0.001	2.68	2.02–3.57	< 0.001
Restart after 12 months	38	79.2	4.68	2.32–9.41	< 0.001	-	-	-
Transfer in	125	85.0	6.99	4.43–11.05	< 0.001	3.12	1.92–5.08	< 0.001

PrEP, pre-exposure prophylaxis; OR, odds ratio; aOR, adjusted odds ratio; Ref, reference group.

† , During COVID-19: 2380 (45%).

### Factors associated with retention of pre-exposure prophylaxis medication among clients in Eswatini

A total of 5286 were included in the study; 61% (*n* = 3241) were adhering to PrEP medication. Compared to Facility 2, Facility 1, Facility 3 and Facility 5 had a higher odds patients who returned to PrEP treatment, Facility 1 (aOR: 1.92, 95% CI: 1.58–2.34, *p* < 0.001), Facility 3 (aOR: 2.02, 95% CI: 1.66–2.47, *p* < 0.001) and Facility 5 (aOR: 2.71, 95% CI: 1.18–6.23, *p* = 0.019). Compared to those aged 40 years and above, clients who are aged 15–19 years, 20–24 years, and 25–29 years had lower odds of retention to PrEP medication. Regarding gender, females had higher odds of retention in PrEP medication (OR: 1.50, 95% CI: 1.32–1.71, *p* < 0.001) compared to males; however, the association was no longer statistically significant after adjusting for potential confounders.

Clients who initiated PrEP after the COVID-19 period had higher odds of retention in PrEP medication (aOR: 2.96, 95% CI: 2.58–3.39, *p* < 0.001) compared to those that were initiated during the COVID-19 period; even after adjusting for potential confounders, the association remained statistically significant (see Table 4).

**TABLE 4:** Factors associated with pre-exposure prophylaxis retention among clients in Eswatini.

Variables	Retention status†		Bivariate			Multiple logistic		
	*n*	%	OR	95%CI	*p*	aOR	95%CI	*p*
**Facility name**								
Facility 2	395	49.2	Ref	Ref	-	-	-	-
Facility 1	1579	65.8	1.98	1.69–2.33	< 0.001	1.92	1.58–2.34	< 0.001
Facility 3	1104	63.3	1.77	1.50–2.11	< 0.001	2.02	1.66–2.47	< 0.001
Facility 4	135	45.0	0.85	0.65–1.10	0.215	1.04	0.77–1.41	0.791
Facility 5	28	75.7	3.21	1.50–6.90	0.003	2.71	1.18–6.23	0.019
**Age category (years)**								
40 and above	421	58.0	Ref	Ref	-	-	-	-
15–19	379	63.5	1.26	1.01–1.57	0.042	0.59	0.45–0.77	< 0.001
20–24	863	64.3	1.31	1.08–1.57	0.005	0.64	0.51–0.80	< 0.001
25–29	736	62.4	1.20	0.99–1.45	0.057	0.71	0.57–0.88	0.002
30–39	842	58.4	1.02	0.85–1.22	0.844	-	-	-
**Gender**								
Male	651	22.9	Ref	Ref	-	-	-	-
Female	2590	77.1	1.50	1.32–1.71	< 0.001	-	-	-
**COVID-19 period**								
During COVID-19	1134	47.7	Ref	Ref	-	-	-	-
After COVID-19	2107	72.5	2.90	2.58–3.25	< 0.001	2.96	2.58–3.39	< 0.001
**Population type**								
High-risk males (30–34 years)	147	52.5	Ref	Ref	-	-	-	-
Adolescent girls and young women (16–24 years)	1082	64.7	1.66	1.29–2.14	< 0.001	-	-	-
**Initiation type**								
Transfer in	76	51.7	Ref	Ref	-	-	-	-
First initiation	2624	61.3	1.48	1.07–2.06	0.019	-	-	-
Restart less than 12 months	204	54.1	1.10	0.75–1.61	0.619	-	-	-
Restart after 12 months	28	58.3	1.31	0.68–2.53	0.424	-	-	-

PrEP, pre-exposure prophylaxis; OR, odds ratio; aOR, adjusted odds ratio; Ref, reference group.

† , Retained in PrEP 3241 (61.3%).

## Discussion

In this study, we found that PrEP uptake was generally low during the COVID-19 period because there were many interruptions in people’s movement because of travel restrictions.

During the COVID-19 period, we can see that most initiations were from the ages 30 – 39, which is the group with the highest prevalence in the country according to the Population-based HIV Impact Assessment (PHIA) project (2021).^18^ One of the reasons that might have caused this group to have a high number of initiations may be the fact that the group is sort of free from parental guidance.

The study also indicates that the highest number of initiations in the facilities was reported in Facility 3 with 32% of initiations. One may think that the facility is a bit rural and situated next to a university campus, surrounding farms, and huge factories. On the other hand, the high initiation in the rural areas shows the level of compliance with the travel restrictions as towns were guarded by the army and police.

There was a change during the COVID-19 pandemic as there were high initiations in Facility 1.^16^ This facility is closer to town and is in a semi-urban place where low-income earners reside. It was also observed that there was a 15.3% increase in initiations among females, while a 13.3% decrease among males after COVID-19.

With the results of this study, we can concur with what Hartnett et al. (2020) stated that major changes in the utilisation of healthcare services occurred during the COVID-19 period. They further mention that the lockdowns and stay-at-home orders were forcing low uptake of healthcare services. We also see a reduction in services uptake during the period in the study.

On the other hand, Kerzner et al. (2022) showed a significant increase in PrEP uptake.^6^ The study reported that innovative actions such as PrEP campaigns, development, and distribution of COVID-19 PrEP information and virtual engagements resulted in increased uptake of PrEP services during the COVID-19 period in countries such as Kenya (52.2% increase), Uganda (142.2% increase) and South Africa (201.6% increase).^6^ This was because of concerted efforts of the government and implementing partners during the COVID-19 pandemic, which yielded tangible results. Kerzner’s study indicates that although there was an increase in PrEP initiations (uptake), the countries experienced a decline in new PrEP users (first-time initiations), especially in countries such as the Dominican Republic (8.6%), Lesotho (5.2%), Thailand (11.3%), and Ukraine (14.4%).^6^ Such results show that the clients who were initiated in the COVID-19 period were re-initiations, people who have the information and used the prophylaxis before. Most countries supported by the US President’s Emergency Plan for AIDS Relief (PEPFAR) adapted well during the COVID-19 pandemic in those in the continuity of HIV services provision; however, other countries lagged.^17^

### Limitations

Inadequate documentation of PrEP uptake and continuation before COVID-19 is a potential limitation. Pre-exposure prophylaxis rollout began in Eswatini in 2018 and COVID-19 started in 2019, during the service scaling-up phase. The low number of facilities offering PrEP at the pandemic’s onset likely affected the numbers. During COVID-19, a significant increase in PrEP initiation among female clients was observed, which continued post-pandemic. Factors contributing to this rise included scaling up PrEP to all HIV-negative pregnant women attending antenatal care (ANC) and improved marketing efforts.

## Conclusion

This study concludes that COVID-19 significantly influenced the uptake and retention of PrEP services in Eswatini. The pandemic disrupted both the initiation and continuity of these services. The findings provide critical insights for PrEP programme planning and positioning in response to pandemics, underscoring the necessity for flexible and innovative healthcare delivery approaches, such as targeted campaigns, to mitigate service disruptions during public health crises. In addition, the study highlights the need for tailored interventions to address changing PrEP uptake patterns across different demographic groups and acknowledges the potential for re-initiations among previously exposed individuals. These results emphasise the importance of maintaining continuity in HIV prevention efforts amid broader public health challenges, showcasing the essential role of responsive healthcare systems in safeguarding community health and well-being.

The observed decrease in PrEP uptake among men post-COVID-19 suggests the need for expanding PrEP options. Other countries have implemented Event-Driven PrEP (ED PrEP), where men take PrEP only when anticipating exposure to HIV. Eswatini should consider investigating this option to enhance prevention, particularly among men. The study found that females had higher odds of adhering to PrEP medication (OR: 1.50, 95% CI: 1.32–1.71) compared to males. Enhancing and diversifying PrEP options for men could significantly improve HIV prevention in the country.
